# Neutral sphingomyelinase inhibition promotes local and network degeneration in vitro and in vivo

**DOI:** 10.1186/s12964-023-01291-1

**Published:** 2023-10-30

**Authors:** Michael L. Risner, Marcio Ribeiro, Nolan R. McGrady, Bhanu S. Kagitapalli, Xitiz Chamling, Donald J. Zack, David J. Calkins

**Affiliations:** 1 Department of Ophthalmology and Visual Sciences, Vanderbilt Eye Institute, Vanderbilt University Medical Center, AA7103 MCN/VUIIS, 1161 21st Ave S., Nashville, TN 37232 USA; 2 Present Address: Department of Foundational Medical Studies, Eye Research Center, Oakland University William Beaumont School of Medicine, 369 Dodge Hall, 118 Library Dr., Rochester, MI 48309 USA; 3 Department of Ophthalmology, Wilmer Eye Institute, Johns Hopkins University School of Medicine, Baltimore, MD 21287 USA

**Keywords:** Human embryonic stem cells, Retinal ganglion cells, Extracellular vesicles, Apoptosis, Neutral sphingomyelinase, GM1 ganglioside, Ceramide, Mitochondria

## Abstract

**Background:**

Cell-to-cell communication is vital for tissues to respond, adapt, and thrive in the prevailing milieu. Several mechanisms mediate intercellular signaling, including tunneling nanotubes, gap junctions, and extracellular vesicles (EV). Depending on local and systemic conditions, EVs may contain cargoes that promote survival, neuroprotection, or pathology. Our understanding of pathologic intercellular signaling has been bolstered by disease models using neurons derived from human pluripotent stems cells (hPSC).

**Methods:**

Here, we used hPSC-derived retinal ganglion cells (hRGC) and the mouse visual system to investigate the influence of modulating EV generation on intercellular trafficking and cell survival. We probed the impact of EV modulation on cell survival by decreasing the catabolism of sphingomyelin into ceramide through inhibition of neutral sphingomyelinase (nSMase), using GW4869. We assayed for cell survival in vitro by probing for annexin A5, phosphatidylserine, viable mitochondria, and mitochondrial reactive oxygen species. In vivo, we performed intraocular injections of GW4869 and measured RGC and superior colliculus neuron density and RGC anterograde axon transport.

**Results:**

Following twenty-four hours of dosing hRGCs with GW4869, we found that inhibition of nSMase decreased ceramide and enhanced GM1 ganglioside accumulation. This inhibition also reduced the density of small EVs, increased the density of large EVs, and enriched the pro-apoptotic protein, annexin A5. Reducing nSMase activity increased hRGC apoptosis initiation due to enhanced density and uptake of apoptotic particles, as identified by the annexin A5 binding phospholipid, phosphatidylserine. We assayed intercellular trafficking of mitochondria by developing a coculture system of GW4869-treated and naïve hRGCs. In treated cells, inhibition of nSMase reduced the number of viable mitochondria, while driving mitochondrial reactive oxygen species not only in treated, but also in naive hRGCs added in coculture. In mice, 20 days following a single intravitreal injection of GW4869, we found a significant loss of RGCs and their axonal recipient neurons in the superior colliculus. This followed a more dramatic reduction in anterograde RGC axon transport to the colliculus.

**Conclusion:**

Overall, our data suggest that perturbing the physiologic catabolism of sphingomyelin by inhibiting nSMase reorganizes plasma membrane associated sphingolipids, alters the profile of neuron-generated EVs, and promotes neurodegeneration in vitro and in vivo by shifting the balance of pro-survival versus -degenerative EVs.

Video Abstract

**Supplementary Information:**

The online version contains supplementary material available at 10.1186/s12964-023-01291-1.

## Background

Cells communicate with neighboring and distant cells through paracrine signaling via extracellular vesicles (EV). EVs are phospholipid bilayer membrane bound packets, containing nucleic acids, proteins, lipids, and organelles [1]. EVs can invade and deploy cargo into recipient cells and alter their phenotype [2]. Under physiologic conditions, EVs envelop cell debris for clearance, and their cargoes can shape neuronal axon guidance, myelination, provide trophic support, enhance synaptic activity, and promote regeneration [3–9]. However, pathologic cells may transfer misfolded and aggregate proteins and disease-related nucleic acids via EVs to neighboring cells, promoting degeneration [10]. Based on this evidence, EVs and their cargoes are targets for neuroprotective and regenerative therapies for neurodegenerative diseases.

The goal of devising EV-based treatments for neurodegenerative diseases is challenged because several EV types have been distinguished based on size, cargoes, and mechanisms for biogenesis [1]. Primary EV types include exosomes, microvesicles, and apoptotic bodies. A commonality among exosomes, microvesicles, and apoptotic bodies is that their biogenesis appears to be dependent on the presence of a specific plasma membrane associated lipid, ceramide [11–13]. Ceramide concentration within plasma membranes can be locally altered via the hydrolysis of sphingomyelin by neutral sphingomyelinase (nSMase, also known as sphingomyelin phosphodiesterase). Activation of nSMase alters plasma membrane associated lipid and protein profiles, producing apoptotic bodies through membrane scission [14]. Inhibition of nSMase reduces the formation of exosomes by limiting the ceramide-dependent pathway but increases the formation of microvesicles [11, 12]. Taken together, the metabolism of sphingomyelin into ceramide by nSMase significantly affects plasma membrane associated lipids and intercellular signaling via EVs [13]. The influence of the action of nSMase on plasma membrane associated lipids and EV signaling in the context of cell viability is central to understanding local and network pathophysiology during stress.

Previous investigations suggest stem cell derived exosomes provide protection against progression of neurodegenerative diseases, including glaucomatous optic neuropathy (glaucoma) [15–17]. Glaucoma is an age-related neurodegenerative disease that causes irreversible vision loss by targeting retinal ganglion cells (RGC) and their axons for degeneration. Early pathological events underlying glaucoma include signatures of axonopathy, identified by the loss of anterograde and retrograde RGC axonal transport [18–21]. Deficits in anterograde and retrograde RGC axonal transport decrease transfer of essential proteins and organelles to distal axons and brain-derived growth factor (BDNF) to RGCs needed to maintain communication between the retina and brain [18, 22–24]. In rodent models of glaucoma, evidence suggests that exosomes derived from mesenchymal stem cells protect against axon degeneration [15, 16]. Based on this evidence, harnessing EV biogenesis appears to be a potential neuroprotective strategy for glaucoma.

Here, we tested the influence of reducing exosome generation in vitro, using human pluripotent stem cell (hPSC)-derived retinal ganglion cells (hRGCs), and in vivo, employing the mouse visual system. We reduced exosome biogenesis through the ceramide-dependent pathway, using the nSMase inhibitor, GW4869 [25]. As expected, inhibition of nSMase decreased ceramide, but also increased GM1 ganglioside, which is a complex sphingolipid localized to RGCs that potentiates BDNF signaling [21, 26, 27]. As anticipated, nSMase inhibition decreased the production of small EVs, but also enhanced the generation of larger EVs expressing annexin A5 (ANXA5, apoptotic bodies). We provide evidence that the increased density of large EVs and interactions with viable cells promotes degeneration. Using a coculture strategy consisting of cells treated with GW4869 and naïve cells, we observed inhibition of nSMase reduced the quantity of viable mitochondria in treated cells and increased accumulation of mitochondrial reactive oxygen species in both treated and naïve cells. Finally, we found GW4869 reduced the density of RGCs and their target neurons in the superior colliculus (SC) along with degradation of RGC anterograde axonal transport to the SC. Our findings indicate that perturbing the physiologic catabolism of sphingomyelin by targeting nSMase reconfigures plasma membrane associated sphingolipids and promotes neurodegeneration in vitro and in vivo by tipping the physiologic equilibrium of the extracellular particle environment toward degeneration.

## Materials and methods

### Animals

All animal procedures were approved by the Vanderbilt University Institutional Animal Care and Use Committee and were conducted in accordance with the Association for Research in Vision and Ophthalmology (ARVO) Statement for the Use of Animals in Ophthalmic and Vision Research. C57BL/6 male mice, aged 45 to 60 d, were purchased from Charles River Laboratories (027, Wilmington, MA). Mice were housed at the Vanderbilt Division for Animal Care under a 12 h light 12 h dark cycle and provided water and standard rodent chow as desired.

### In vivo pharmacology and neural tracing

We injected either saline, DMSO (2.89%, 1.5 µL), or GW4869 (10 µM, 1.5 µL, D1692, Millipore-Sigma, Burlington MA) dissolved in DMSO (2.89%) into the vitreous body of eyes using a small-volume syringe and needle in anesthetized mice (2.5% isoflurane). We did not notice the formation of any corneal or lens opacities following the procedure. Some animals were sacrificed 1 d after injections for immunohistochemistry. For the remainder of the animals, we determined the influence of prolonged exposure to GW4869 (20 d). Two days prior to the 20-d endpoint, we injected 1% cholera toxin subunit b 488 (CTB, 1.5 µL, C34775, Invitrogen, Waltham, MA) into the eye near the ora serrata to track RGC uptake and anterograde axon transport of CTB to the SC. At the experimental endpoint, animals were sacrificed and perfused as described below. Whole retinas and brains were dissected out and fixed in 4% PFA. We then dissected out the SC and performed coronal serial sections as described previously [19–21, 28].

### Cell line and culture conditions

H9 hESCs were obtained from WiCell (WA09, Madison, WI). H9 BRN3B-P2A-tdTomato-P2A-THY1.2 hESCs were generated as previously described [29]. Following genome editing and proliferation, H9 BRN3B-P2A-tdTomato-P2A-THY1.2 hESCs were chemically induced toward a RGC fate by the addition and subtraction of small molecules over 40 d [29]. After chemical differentiation and immunopurification by targeting THY1.2, cells were aliquoted at a density of 1 × 10^6^ to 5 × 10^6^ cells / mL of CryoStor (CS10, Millipore-Sigma). Cells were shipped overnight on dry ice from Johns Hopkins University School of Medicine to Vanderbilt University Medical Center and immediately stored in liquid nitrogen until use.

H9 BRN3B-P2A-tdTomato-P2A-THY1.2 hESCs differentiated toward a RGC lineage (hRGC) were prepared for cultured as described in an earlier report [30]. At the time of use, hRGCs were thawed by gently swirling the vial in a 37 °C water bath for 1 min, and the vial was disinfected with 70% ETOH. One mL of thawed hRGCs were resuspend in 6 mL culture medium consisting of 1:1 DMEM/F12 (10,565,018, Gibco, Waltham, MA) and Neurobasal medium (21,103,049, Gibco) and 2% B27 supplement (A1486701, Gibco), 1% N2 supplement (17,502,048, Gibco), and 1% gentamicin (15,710,064, Gibco). The cell suspension was centrifuged at 150 × g for 5 min. The supernatant was removed, and the cell pellet was resuspended in fresh culture medium. hRGCs were plated on 4% HCl-washed borosilicate glass coverslips coated with poly-d-lysine (50 µL/mL, 24 h, 37 °C, A003E, Millipore-Sigma) and laminin (10 µL/mL, 4 h, 37 °C, CB40232, Corning, Corning, NY) at a density of 6 × 10^4^ cells / cm^2^. Cell cultures were maintained at 5% CO_2_ 37 °C.

We determined the influence of inhibiting nSMase activity by treating samples with GW4869 (5 to 10 µM) for 20 to 24 h. GW4869 working solutions contained 2.89% DMSO; therefore, for most control experiments we added 2.89% DMSO for 20 to 24 h. We assayed the impact of inhibiting acid sphingomyelinase (aSMase) action by treating cells with 10 µM desipramine for 24 h. Desipramine stocks were made using double deionized water and working concentrations were produced by adding stock to culture medium. In some experiments we tested the influence of DMSO on outcome measurements, adding fresh medium at the time of treatment (control). In a subset of experiments, we measured initiation of apoptosis using pSIVA (15 µL/mL, APO004, Bio-Rad Laboratories, Hercules, CA), following the directions provided by the manufacturer. For a few experiments, we compared pSIVA versus Propidium Iodide (10 µL/mL, Bio-Rad Laboratories) fluorescence following administration of 10 µM GW4836 in cultured Adult Retinal Pigmented Epithelial cells (ARPE-19, CRL-2302, ATCC, Manassas, VA).

In a subset of experiments, we determined the influence of GW4869 on intercellular trafficking of mitochondria and CTB using a coculture strategy. One group of cells were plated at a density of 3 × 10^4^ cells / cm^2^. The following day, we treated cells with either GW4869 or DMSO for 20 to 24 h. Afterwards, we exchanged the drug conditioned medium twice with fresh medium. After the last medium exchange, we added 0.5% CTB-488 and 300 nM MitoTracker Deep Red (M22426, Invitrogen) and incubated at 37 °C for 1 h. Following incubation, we performed two fresh medium exchanges to remove residual CTB and MitoTracker. Then, we plated a second group of naïve cells at a density of 3 × 10^4^. The following day, we prepared samples for live imaging. In a small number of experiments, we confirmed mitochondria and CTB labeling, using CellLight Mitochondria-GFP Bacmam (C10600, Invitrogen) and CTB-647 (C34778, Invitrogen).

In a different set of experiments, we employed a similar coculture approach to measure mitochondrial reactive oxygen species. Again, we plated one group of cells at a density of 3 × 10^4^ cells / cm^2^. The next day, we treated samples with either DMSO or GW4869. Following 20 to 24 h of drug treatment, we performed two fresh medium exchanges, and then incubated cells in 0.5% CTB-488 for 1 h. After incubation, we performed another two medium exchanges to remove residual CTB-488. Then, we plated a second group of naïve hRGCs at a density of 3 × 10^4^ cells / cm^2^. The next day, we incubated the coculture in mtSOX Deep Red (10 µm/L, MT14, Dojindo Laboratories, Kumamoto, Japan) for 30 min and performed live imaging.

### Immunocytochemistry, immunohistochemistry, and imaging

Immunocytochemistry was performed as previously described [30, 31]. Cells were fixed for 30 min in 4% paraformaldehyde. Afterwards, cells were incubated in a blocking solution containing 5% normal donkey serum, 0.1% Triton-X, and DPBS for 2 h at room temperature. After blocking, we used the following primary antibodies: nSMase2 (1:200, ab85017, Abcam, Cambridge UK), GM1 ganglioside (1:200, ab23943, Abcam), ceramide (1:100, C8104, Millipore-Sigma). We incubated cells in blocking solution plus primary antibodies overnight at 4 °C. The following morning, we washed cells 3 times with DPBS, and then, incubated cultures in appropriate secondary antibodies (Jackson ImmunoResearch Laboratories, West Grove, PA) for 2 h at room temperature. In a subset of experiments, we confirmed ceramide labeling using C6-NBD Ceramide (10 µM, 810209P, Avanti Polar Lipids, Alabaster, AL).

Immunohistochemistry was performed as previous described [32]. At the time of use, mice were transcardially perfused with PBS followed by PFA (4%). Whole retinas were dissected from the eye and post fixed in 4% PFA for 2 h at room temperature. After rinsing PFA, retinas were incubated in a 30% sucrose gradient for 3 d followed by 3 freeze–thaw cycles at -80 °C-room temperature. Afterwards, tissues were blocked (5% normal donkey serum, 0.1% Triton-X) for 2 h at room temperature. Then, retinas were incubated in blocking solution plus primary antibodies against nSMase2 (1:200, ab85017, Abcam), RNA-binding protein with multiple splicing (RBPMS, 1:500, GTX118619, GeneTex, Irving, CA), POU class 4 homeobox 1 (Brn3a, 1:200, AB5945, Millipore-Sigma), NeuN (1:500, 12,943, Cell Signaling Technologies, Danvers, MA) for 3 d at 4 °C. After incubating in primary antibodies, retinas were rinsed with PBS and incubated in appropriate secondary antibodies (Jackson ImmunoResearch Laboratories) for 2 h at room temperature. Finally, retinas were mounted onto slides and coverslipped with Fluormount G. All imaging was performing using a Nikon spinning disk confocal microscopy at the Vanderbilt University Nikon Center for Excellence as described earlier [31]. Laser line intensity and exposure time were kept constant for each dependent variable between conditions.

### Image analysis

We measured mean fluorescence intensity (intensity) for each dependent variable (e.g. nSMase2, GM1 ganglioside, MitoTracker, mtSOX) by outlining cell profiles, using the freehand draw function in ImageJ (Version 1.53t, NIH, Bethesda, MD). We performed colocalization analysis in ImageJ, using the Costes’ threshold Mander’s coefficient method as previously described [33]. For mouse retinas, we measured nSMase2 and CTB mean intensity in the RGC layer, indicated by RBPMS or Brn3a labeling. We measured RGC density and SC neuron density using similar methods previously described [19, 28]. We quantified RGC axon transport of CTB to the SC using methods previously described [19–21, 28, 32]. For representative graphics, we typically used the background subtraction, enhance contrast, and despeckle functions provided in ImageJ. During image analysis, investigators were not blinded regarding experimental condition.

### Extracellular vesicle isolation and particle analysis

For isolation of extracellular vesicles, cells were grown to 70–80% confluency. Growth media was removed and then replaced with growth media containing 10 µM GW4869 or growth media containing DMSO (2.89%, vehicle). After 20–24 h, culture media was collected and subjected to differential centrifugation as described previously [34]. After each step, supernatant was transferred to a new tube. Briefly, collected media was sequentially centrifuged for 10 min at 300 × g to remove any cells, and 25 min 2000 × g to remove cell debris using a tabletop centrifuge. Supernatant was then centrifuged for 30 min at 10,000 × g MLA55 ultracentrifuge rotor (Beckman Coulter, Brea, CA) to remove microvesicles. Supernatant was then centrifuged at for 18 h at 100,000 × g in a Type 45 Ti rotor (Beckman Coulter) to obtain small EVs. Small EV-containing pellets were resuspended in 200 µL sterile cold PBS and then re-pelleted at 100,000 × g for 4 h using a TLA55 rotor (Beckman Coulter) and resuspended in 50 µL sterile PBS. Nanoparticle tracking (ZetaView, Particle Metrix, Ammersee, Germany) was used to determine EV size and number.

### Western blot

For western blot analysis, small EVs were isolated as described above. After centrifugation at 100,000 × g, pellets were resuspended in 2% SDS. Protein concentration was determined using a Pierce BCA Protein Assay Kit (23,235, Thermo Fisher Scientific, Waltham, MA). Small EV samples were loaded equally (10 µg) in 4–20% gradient SDS-PAGE gels (Bio-Rad Laboratories) and then transferred overnight at 4 °C to PVDF membranes (Millipore-Sigma). Membranes were first blocked using 5% BSA in PBS with Tween (PBST) for 1 h at room temperature. The primary antibody (Annexin A5, 1:200, AF399, R&D Systems) was diluted in 1% BSA in PBST and incubated overnight at 4 °C. AlexaFluor conjugated secondary antibody (Cy647, Jackson ImmunoResearch Laboratories) was diluted 1:1000 in 5% BSA in PBST and incubated for 2 h at room temperature. Membranes were imaged using a ChemiDoc MP imaging system (Bio-Rad Laboratories).

### Data analysis

We used GraphPad Prism 9 (GraphPad Software, San Diego, CA) for statistical analyses. All data are reported as mean ± standard error of the mean (SEM). We performed outlier analysis (Grubb’s test) and normality test (D’Agostino-Pearson) for each dataset prior to statistical comparison. We used parametric statistical test for datasets meeting the criteria for normality, and non-parametric statistics for datasets that failed normality test. We have identified each statistical test in the figure legends.

## Results

**Fig. 1:**
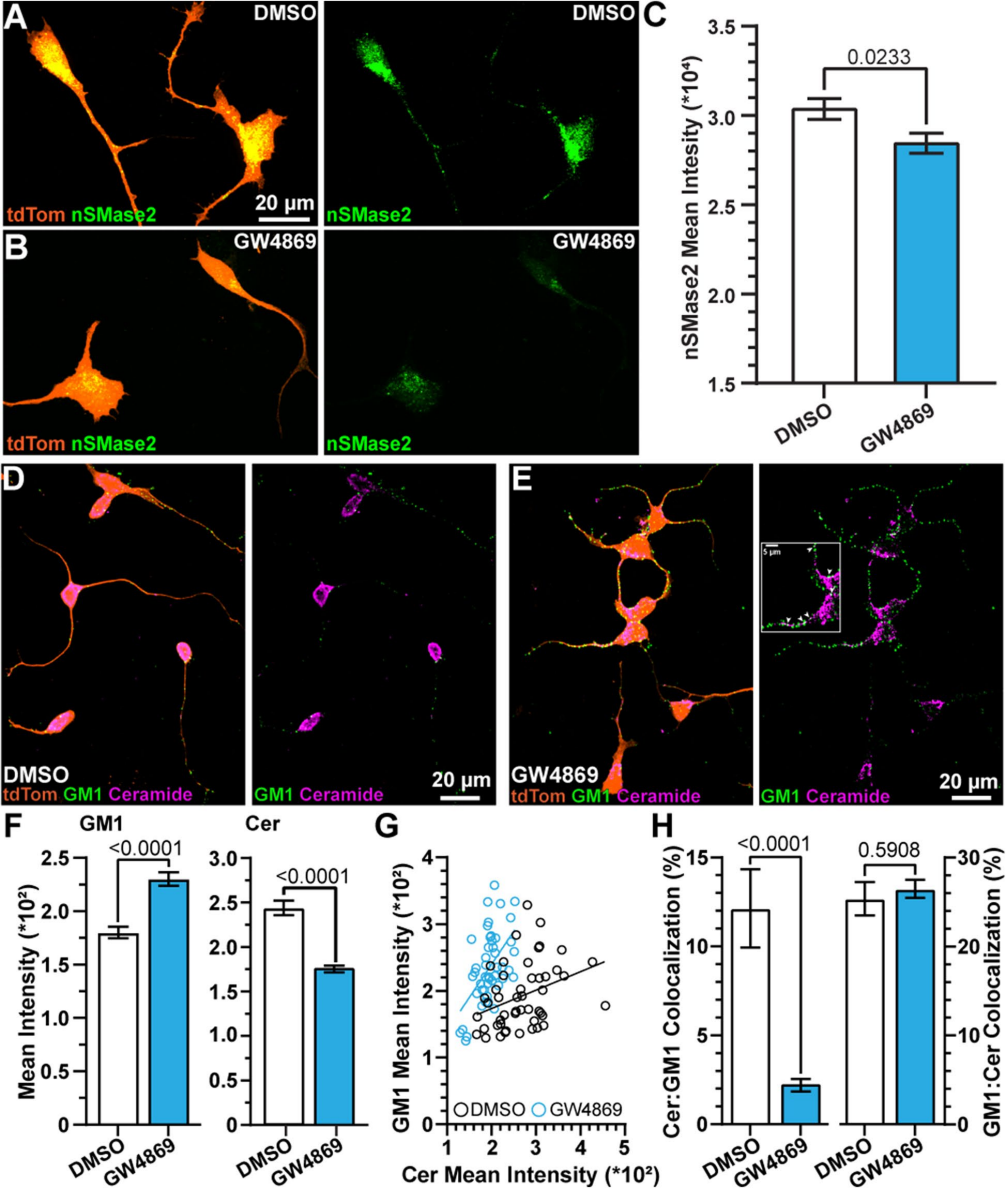
Inhibition of nSMase alters plasma membrane-associated lipids. **A**, **B** Example confocal images of (**A**) DMSO- and (**B**) GW4869-treated 3 DIV hRGCs expressing tdTomato (orange) immunolabeled against nSMase2 (green). (**C**) GW4869 reduced nSMase2 immunofluorescence in hRGC cultures (*p* = 0.0233). *N* = 4 independent samples of 112 DMSO-treated cells (95 images) and 118 GW4869-treated cells (88 images). **D**, **E** Images of fixed (**D**) DMSO- and (**E**) GW4869-treated hRGCs immunolabeled against GM1 ganglioside (green) and ceramide (magenta). Inset indicates points of overlapping signals from GM1 and ceramide (arrowheads). (**F**, left) GW4869 significantly increased GM1 ganglioside (DMSO: *N* = 125 cells across 110 images from 4 replicates; GW4869: *N* = 109 cells across 90 images from 5 replicates). (**F**, right) GW4869 decreased ceramide (Cer) localization to hRGCs (*p* < 0.001). DMSO: *N* = 48 cells across 26 images from 2 replicates; GW4869: 47 cells across 32 images from 2 replicates. **G** There is a positive relationship between GM1 and Cer immunolabeling intensity that is altered by GW4869 (DMSO: *p* = 0.0139, *R*^2^ = 0.12, GW4869: *p* < 0.001, *R*^2^ = 0.29). DMSO *N* = 48 cells from 2 replicates (26 images). GW4869 *N* = 47 cells across 2 replicates (32 images). **H** The percent of colocalization (Mander’s) between ceramide and GM1 immunolabeling is reduced by GW4869 (left, *p* < 0.001) but the percent of colocalization between GM1 and ceramide remained unaltered (right, *p* = 0.5908). DMSO *N* = 48 cells from 2 replicates. GW4869 *N* = 47 cells across 2 replicates. Statistics: **C**, **H** Mann–Whitney test, (**F**) t-test, (**G**) Linear regression. Data presented as mean ± sem

We tested the impact of incubating hRGCs in GW4869 (10 µM) or DMSO (2.89%) for 24 h on nSMase2 protein expression by immunolabeling [35]. We observed GW4869 degraded nSMase2 immunolabeling in hRGCs (Fig. 1A, B). Quantification of the mean intensity of nSMase2 immunofluorescence indicated GW4869 significantly reduced protein expression by 12% compared to DMSO-treated cells (*p* = 0.0233, Fig. 1C). We confirmed immunolabeling of nSMase2 in vitro by incubating cells only in secondary antibody. When only secondary antibody was included, immunofluorescence was muted and appeared randomly localized (Fig. S1A, B). Moreover, we tested and the influence of GW4869 on nSMase2 immunofluorescence in RGCs in whole-mount retinas of mice. Similar to a previous report [36], we observed nSMase2 labeling in the RGC layer, identified by immunoreactivity against RBPMS, and we found 24 h after intravitreal injection of GW4869, nSMase2 accumulation in the RGC layer significantly diminished by 30% (*p* = 0.0444, Fig. S2A, B).

Inhibition of SMase activity reduces the catabolism of sphingomyelin into phosphocholine and ceramide, which forms the transmembrane portion of the complex sphingolipid, GM1 ganglioside [37–39]. Therefore, we tested the influence of GW4869 on ceramide and GM1 ganglioside content in hRGCs. In DMSO-treated cultures, GM1 labeling appeared punctate, collecting mostly along hRGC neurites (Fig. 1D). Ceramide seemed to accumulate mostly in the soma with additional fluorescence sparsely localized along neurites (Fig. 1D). We confirmed localization of ceramide, using C6-NBD ceramide (Fig. S1C). Following treatment with GW4869, GM1 puncta appeared larger and densely localized within hRGC neurites, and ceramide labeling appeared fragmented (Fig. 1E). Quantification of the mean intensity produced by GM1 ganglioside and ceramide labeling indicated GW4869 significantly increased GM1 by 28% and significantly reduced ceramide fluorescence by 27% compared to DMSO-treated cells (*p* < 0.001, Fig. 1F). Similarly, one day after an intraocular injection of GW4869, we found GM1 significantly increased by 12% in the RGC layer in whole-mount retinas compared to DMSO-treated control retinas (*p* = 0.0237, Fig. S2C, D).

Using data from a subset of cultures, we directly compared GM1 versus ceramide labeling cell-by-cell. For DMSO-treated cells, we observed a positive correlation between GM1 and ceramide (*p* = 0.0139, *R*^2^ = 0.12, Fig. 1G). Following treatment with GW4869, the positive correlation between GM1 and ceramide modestly increased (*p* < 0.001 *R*^2^ = 0.3, Fig. 1G). To further explore this result, we measured the percent of ceramide labeling colocalizing with GM1 and vice versa, using Mander’s coefficients. We found GW4869 significantly reduced the percentage of ceramide fluorescence overlapping with GM1 labeling by 81% (*p* < 0.001, Fig. 1H, left). However, the proportion of GM1 fluorescence colocalizing with ceramide remained unchanged by GW4869 treatment (DMSO = 25%, GW4869 = 26%, *p* = 0.5908, Fig. 1H, right). Given that ceramide and GM1 are not expected to be perfectly correlated because there are several other gangliosides with ceramide moieties, as well as other pools of ceramide, we therefore suspect inhibition of nSMase by GW4869 alters the composition of lipids in the plasma membrane by increasing GM1 ganglioside and diminishing ceramide unassociated with GM1 ganglioside.

**Fig. 2:**
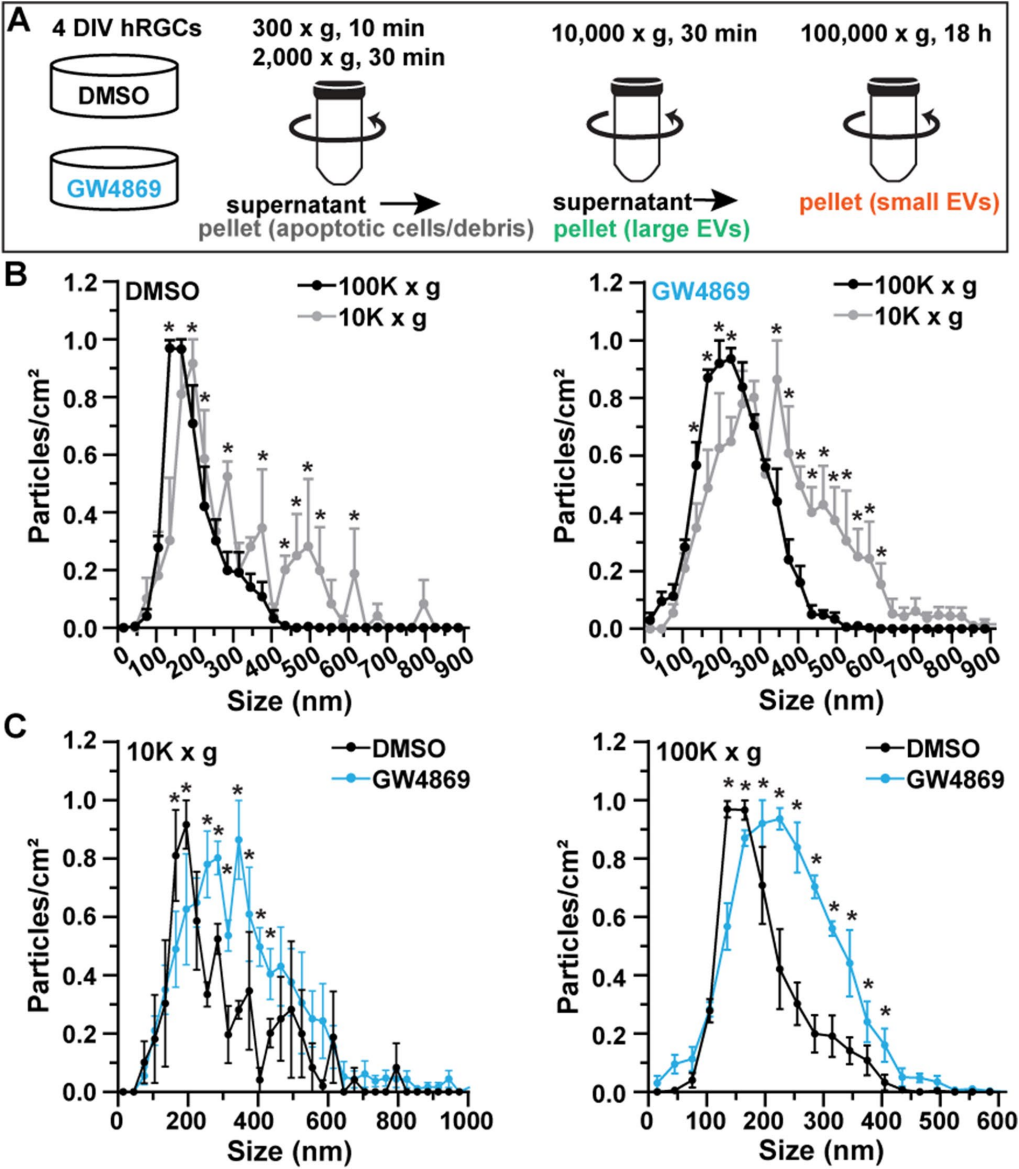
Inhibition of nSMase differentially modulates small and large EVs. **A** Schematic of workflow to obtain 10,000 × g and 100,000 × g sediments from hRGC supernatant. **B** 100,000 × g sediment contain smaller particles versus 10,000 × g sediment in both (left) DMSO- and (right) GW4869-treated cultures. Asterisks indicate *p* ≤ 0.0451. **C** Quantification of particle analysis of 10,000 and 100,000 × g pellets from supernatant of DMSO- and GW4869-treated cells. *N* = 3 independent samples from each condition. Asterisks indicate *p* ≤ 0.0115. Statistics: **B**, **C** Two-way repeated measures ANOVA, Bonferroni post hoc tests. Data presented as mean ± sem

Inhibition of nSMase differentially influences the biogenesis of small and large EVs [11, 12]. Therefore, we reasoned GW4869 would reduce the generation of small EVs and enhance the production of large EVs by hRGCs. Thus, prior to harvesting cells for immunocytochemistry, we collected the supernatant and performed differential centrifugation to obtain extracellular material at 10,000 and 100,000 × g (Fig. 2A). We tested if differential centrifugation separated smaller versus larger particles by particle tracking. Comparing the particle sizes from the 10,000 and 100,000 × g sediments of DMSO-treated cells, we found the peak particle size was 135 nm for the 100,000 × g sediment and 195 nm for the 10,000 × g sediment (Fig. 2B, left). For the GW4869-treated cells, the peak particle size was 225 nm for the 100,000 × g pellet and 345 nm for the 10,000 × g pellet (Fig. 2B, left). Based on these observations, differential centrifugation appears to separate distinct distributions of EV-related material obtained from the supernatant of DMSO- and GW4869-treated cells.

After testing the effectiveness of differential centrifugation on particle separation by size, we assayed the influence of GW4869 by directly comparing the particle distributions within each sediment of DMSO- and GW4869 treated cells. In the 10,000 × g sediment, we found GW4869 significantly reduced the concentration of particles between 165 and 195 nm (*p* < 0.001) and enhanced the number of larger particles, ranging in size from 255 to 435 nm (*p* ≤ 0.0359, Fig. 2C, left). Similarly, for the 100,000 g pellet, we found GW4869 significantly reduced the concentration of smaller particles between 135 to 165 nm (*p* ≤ 0.0115) and increased the concentration of particles between 195 to 405 nm (*p* < 0.0001, Fig. 2C, right). Using the 100,000 × g sediments from DMSO- and GW4869-treated cells, we probed for annexin A5 (ANXA5), a marker for apoptotic vesicles) [1]. Based on Western blot intensities, GW4869 increased ANXA5 by 156% (*p* = 0.0115, Fig. S3). Our results indicate GW4869 degrades nSMase2 and ceramide expression, and this diminishment in nSMase2 and ceramide expression reduces small EVs, increases large EVs, and enhances the presence of apoptotic, ANXA5-expressing, particles in the extracellular medium.

**Fig. 3:**
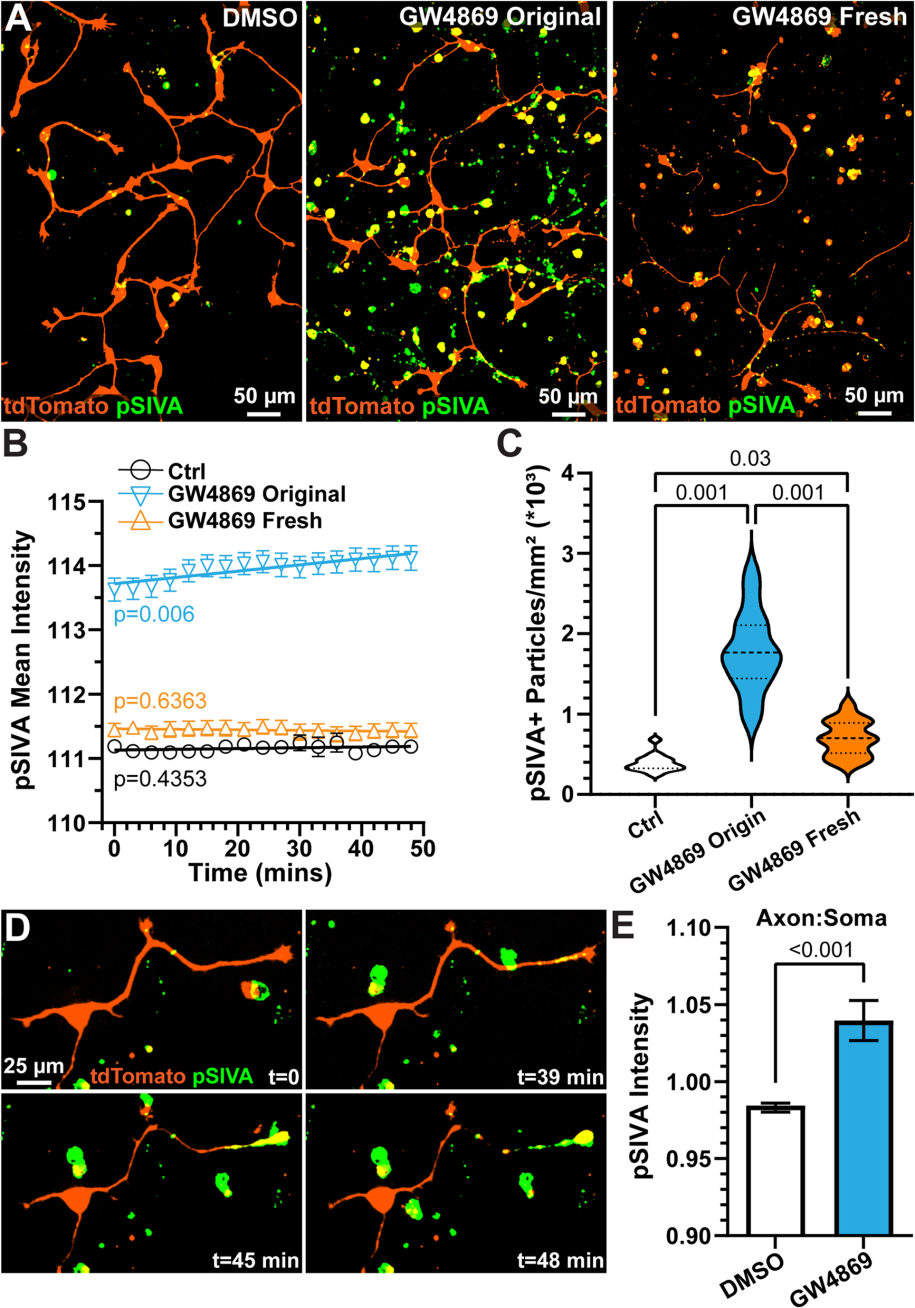
Inhibition of nSMase increases apoptotic particles that promote degeneration. **A** Live spinning disk confocal images of tdTomato-expressing hRGCs (orange) labeled with polarity-Sensitive Indicator of Viability and Apoptosis (pSIVA, green) following treatment with DMSO (left), GW4868 with original medium (middle), or GW4869 with fresh medium (right). **B** Mean intensity of pSIVA over time from control (Ctrl, DMSO or no treatment) cells, cells treated with GW4869 and maintained in original medium, and GW4869-treated cells with fresh medium. DMSO 2 replicates, 15 regions of interest (ROI), GW4869 original medium 4 replicates 18 ROIs, GW4869 fresh medium 2 replicates 15 ROIs. The average intensity of pSIVA plotted over time with linear regression lines of best fit. pSIVA intensity continued to increase in GW4869 original medium cultures (*p* = 0.006). Exchanging original medium with fresh medium reduced pSIVA intensity and halted apoptosis (*p* = 0.6363). pSIVA intensity detected from GW4869 original medium cultures was significantly greater than DMSO and GW4869 fresh medium conditions at each time point (*p* < 0.001). We did not detect a significant difference in pSIVA intensity between control and DMSO conditions (*p* ≥ 0.31). **C** Density of pSIVA-positive particles detected for each condition. GW4869 increased the number of pSIVA-positive particles in both original and fresh medium conditions (*p* ≤ 0.03). Removing the original medium and exchanging fresh medium reduced the number of pSIVA-positive particles in GW4869-treated cells. **D** Example time-lapse images of an hRGC treated with GW4869 and maintained in original medium with a degenerating putative axon. **E** Quantification of pSIVA localized to putative axons relative to somas following 48 min of live imaging. A value of 1 indicates equal intensity in somas and axons. pSIVA intensity was enhanced along the axons of cells treated with GW4869 and maintained in original medium (*p* < 0.001). DMSO: 2 replicates 45 cells. GW4869: 4 replicates 79 cells. Statistics: (**B**) Linear regression. **B** Repeated measures Two-Way ANOVA, Tukey’s post hoc comparisons, (**C**) Kruskal–Wallis test, Dunn’s post-hoc comparisons, (**E**) Mann–Whitney test

Next, we sought to further explore our finding that inhibition of nSMase by GW4869 promotes apoptosis as indicated by increased accumulation of ANXA5-expressing particles in the supernatant (Fig. S3). In control and GW4869-treated samples, we probed for apoptosis initiation by labeling cells with pSIVA (polarity-Sensitive Indicator of Viability and Apoptosis), which is an indicator of extracellular exposure of phosphatidylserine and the binding partner of ANXA5 [40, 41]. For control samples, we noticed a few instances of pSIVA-positive cell bodies and blebs (Fig. 3A, left, Movie 1). Compared to controls, GW4869 appeared to increase degenerated pSIVA-positive somas and blebs, and accumulation in living cells (Fig. 3A, middle). In live samples, we noticed that in GW4869-treated cultures, pSIVA-labeled particles invaded or attached to cells and appeared to induce further degeneration over time (Movies 2 and 3).

In some samples, we sought to determine if exchanging the original medium containing apoptotic particles with fresh medium affected on-going apoptosis. Following a 24 h treatment with GW4869, we performed a medium exchange to remove extracellular apoptotic particles. Then, we reapplied GW4869, treated cells with pSIVA, and performed time-lapse imaging. The medium exchange appeared to decrease the number of pSIVA-positive blebs in the extracellular medium and reduced pSIVA intensity in living cells (Fig. 3A, right). We quantified the influence of GW4869 on apoptosis by measuring the average intensity of pSIVA over time for each region of interest. pSIVA intensity was greatest in GW4869-treated cultures maintained in original medium compared to control and GW4869-treated cultures with fresh medium (*p* < 0.001, Fig. 3B). Moreover, we found a significant positive correlation between pSIVA intensity and time in the GW4869-treated cultures maintained in original medium (*p* = 0.006), indicating increased apoptosis over time. However, this correlation was eradicated when the original medium was exchanged for fresh medium (*p* = 0.6363), suggesting that removing the apoptotic particles from the extracellular medium halted apoptosis. We validated this idea by measuring the number of pSIVA-positive particles for each condition. We found exchanging the medium in the GW4869-treated cultures significantly reduced the density of pSIVA-positive particles by 60% compared to GW4869-treated cells maintained in original medium (*p* = 0.0014, Fig. 3C).

During live imaging of hRGC cultures treated with GW4869 and maintained in original medium, we noticed instances where pSIVA-positive extracellular particles would invade or adhere to a putative axon, inducing an increase in pSIVA fluorescence over time (Fig. 3D, Movies 2 and 3). We sought to determine the direction (i.e., anterograde vs. retrograde) of degeneration by comparing pSIVA intensity along the putative axon versus somatic pSIVA intensity. We found pSIVA significantly accumulated within putative axons relative to the soma in hRGCs treated with GW4869 compared to control samples (*p* < 0.001, Fig. 3E). Finally, we performed experiments relating apoptosis initiation (pSIVA fluorescence) to end-stage apoptosis, using Propidium Iodide (PI), a marker for late apoptosis. We performed these experiments in ARPE 19 cells, revealed by CTB 647 labeling, instead of hRGCs because PI and tdTomato have similar excitation/emission spectra. As anticipated, we found GW4869 first induced an increase in pSIVA followed by an increase in PI in the same cell (Fig. S4). Based on these data, inhibition of nSMase increases the density of pro-apoptotic particles that may interact with living cells, promote degeneration, and apoptosis.

**Fig. 4:**
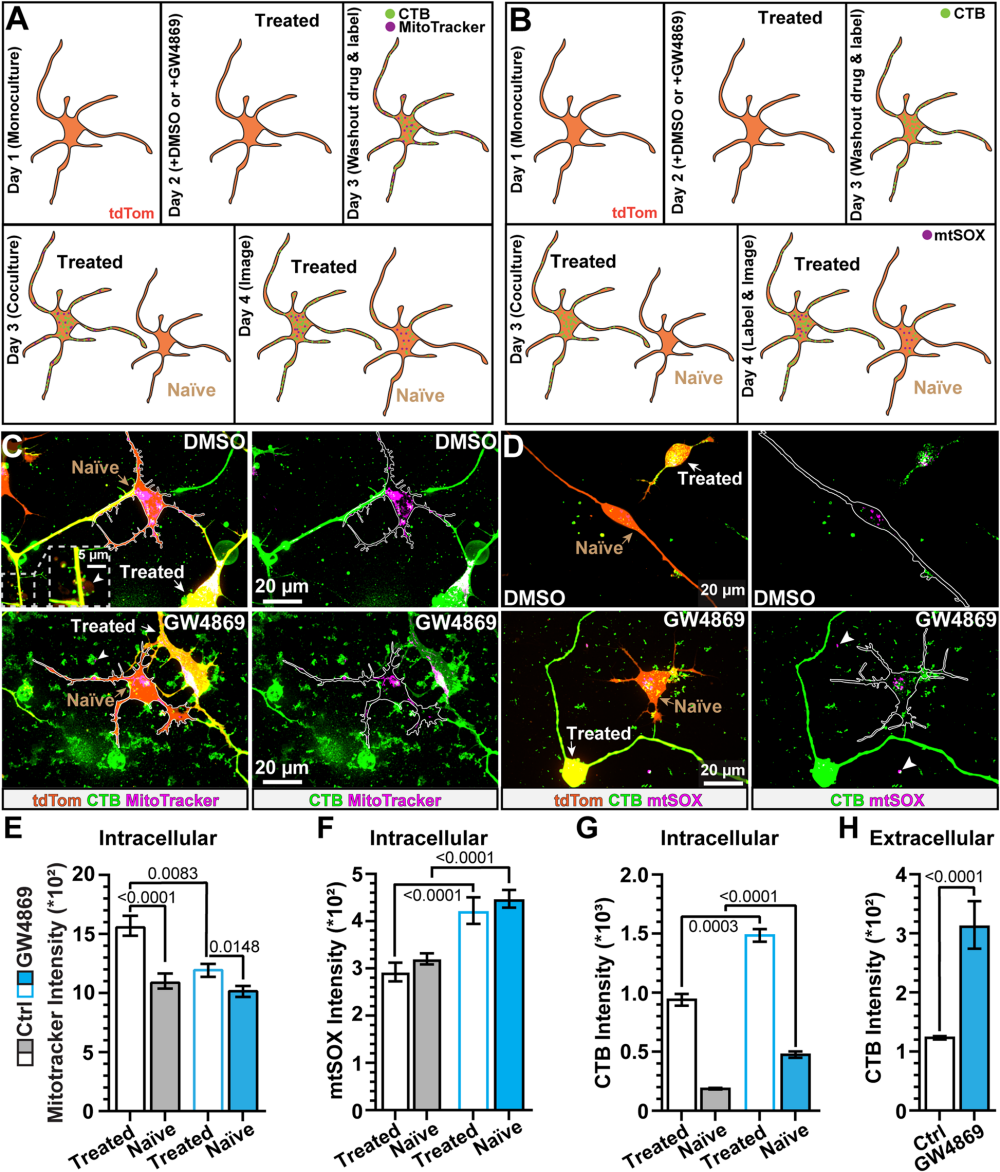
Direct inhibition of nSMase indirectly increases mitochondrial superoxide and CTB uptake in naïve cells. **A** Cartoon of coculture system to detect transfer of CTB and MitoTracker from treated to naïve cells. **B** Diagram of drug treatment, CTB labeling, and mtSOX labeling of hRGC cocultures. **C** Representative images of live tdTomato-positive hRGCs (orange) where control (Ctrl) or GW4869-treated cells were labeled with CTB (green) and MitoTracker (magenta) and naïve cells were not acutely labeled with CTB and MitoTracker. Arrowheads highlight MitoTracker located in the extracellular space. **D** Confocal images of live DMSO- (top) and GW4869-treated hRGCs (bottom) identified by CTB labeling (green) cocultured with naïve cells. Prior to live imaging, cultures were incubated in mtSOX (magenta) to recover degraded mitochondria. Arrowheads indicate mtSOX localized in the extracellular space. **E** GW4869 significantly reduced MitoTracker labeling in treated cells (*p* = 0.0083). 112 Ctrl treated cells and 123 naïve cells from 11 replicates. 81 GW4869 treated cells and 125 naïve cells from 10 replicates. We did not detect a difference in MitoTracker labeling in vehicle and DMSO conditions (*p* ≥ 0.18) so data from both conditions were combined (Ctrl). **F** Quantification of accumulation of mtSOX in treated and naïve cells in Ctrl and GW4869-treated cocultures. GW4869 directly and indirectly (naïve) increased intracellular mtSOX fluorescence intensity compared to respective controls (*p* < 0.001). Ctrl coculture *N* = 32 Ctrl treated cells and 57 naïve cells from 10 replicates. GW4869 coculture *N* = 49 GW4869 treated cells and 80 naïve cells from 7 replicates. We did not detect a significant difference between vehicle and DMSO-treated cocultures, so datasets were combined (*p* ≥ 0.14). **G** Treatment with GW4869 directly (treated) and indirectly (naïve) increased CTB uptake (*p* ≤ 0.003). Ctrl coculture *N* = 84 Ctrl treated cells and 98 naïve cells from 21 replicates. GW4869 coculture *N* = 88 GW4869 treated cells and 91 naïve cells from 12 replicates. CTB uptake by cells in vehicle and DMSO-treated cocultures were similar and data were combined (*p* > 0.9999). **H** GW4869 significantly increased deposition of CTB in the extracellular space (*p* < 0.001). Ctrl *N* = 21 replicates, GW4869 *N* = 12 replicates. Statistics: (C, E, G) Kruskal–Wallis test, Dunn’s post-hoc comparisons, (F, H) *t*-test

Next, we sought to provide additional evidence that inhibiting nSMase promotes apoptosis by measuring mitochondria viability [42]. Furthermore, based on our findings that GW4869 enhanced large EVs and apoptotic bodies, which may harbor intact or fragmented mitochondria, we tested if inhibition of nSMase affects intercellular trafficking of mitochondria [43]. To measure intercellular trafficking of mitochondria, we employed a coculture system of GW4869-treated or control cells identified by acute labeling with cholera toxin subunit B (CTB) and MitoTracker and naïve cells without labeling or drug treatment (Fig. 4A). In separate set of experiments, we also probed for mitochondrial reactive oxygen species (mtSOX), using a similar coculture strategy (Fig. 4B). Based on images from live samples, we could visually distinguish cells heavily labeled by CTB (treated) from cells with weak CTB labeling (naïve, Fig. 4C). Regardless of treatment, in both cocultures, we saw accumulation of MitoTracker within naïve cells, indicating transfer of mitochondria between treated and naïve cells (Fig. 4C, Movies 4 and 5). We confirmed this finding, using a different mitochondria-labeling reagent. Again, we observed intact mitochondria in both treated and naïve cells (Fig. S5). In addition to intracellular mitochondria, we observed mitochondria in the extracellular space in both DMSO- and GW4869-treated cell cultures (Fig. 4C). Application of GW4869, significantly reduced intracellular MitoTracker labeling (*p* = 0.0083, Fig. 4E).

We further tested the notion that inhibition of nSMase promotes initiation of apoptosis by using a similar coculture strategy to probe for mitochondrial reactive oxygen species (mtSOX, Fig. 4B). During live imaging, we noticed sparse accumulation of mtSOX inside of both DMSO-treated cells and fellow naïve cells (Fig. 4D). For cells directly treated with GW4869, mtSOX increased, and this also appeared so for naïve cells in the coculture (Fig. 4D). In addition to mtSOX collecting inside of cells, we also noticed mtSOX in the extracellular space for both conditions (Fig. 4D). We quantified mtSOX accumulation by measuring mean intensity of mtSOX fluorescence separately within cells and in the extracellular space. We found GW4869 significantly enhanced mtSOX fluorescence in both treated (+ 45%) and naïve cells (+ 40%, *p* < 0.0001, Fig. 4F). Furthermore, we found inhibiting nSMase by GW4869 significantly increased mtSOX labeling by 6.5% in the extracellular space (*p* < 0.001). Based on these data, inhibiting nSMase appears to promote trafficking of damaged mitochondria from treated to naïve cells.

Based on our results from GM1 ganglioside labeling (Fig. 1D, E), we anticipated GW4869 would enhance CTB uptake because CTB binds to GM1 ganglioside [44]. In cocultures treated with GW4869, cells appeared more heavily labeled by CTB compared to control cocultures (Fig. 4C, D). Furthermore, we noticed that inhibiting nSMase by GW4869 increased the deposition of CTB-labeled material in the extracellular space (Fig. 4C, D). We quantified intracellular uptake and extracellular deposition of CTB by measuring the mean intensity of CTB fluorescence within treated and naïve cells and in the extracellular space, respectively. As expected, we observed less CTB in naïve cells compared to treated cells independent of condition (*p* < 0.001), proving the effectiveness of the coculture strategy. We found that GW4869 directly enhanced CTB labeling in treated cells (+ 59%, *p* = 0.003) and naïve cells compared to fellow cells in control cocultures (+ 143%, *p* < 0.001, Fig. 3G). Additionally, quantification indicated that GW4869 increased the deposition of CTB-labeled material in the extracellular space (+ 149%, *p* < 0.001, Fig. 4H). We validated that our results were due to the action of nSMase inhibition by also testing the influence of inhibiting aSMase on CTB uptake and transfer. We found that inhibition of aSMase by desipramine does not significantly affect CTB uptake by treated cells (*p* > 0.9999) or CTB migration to naïve cells (*p* = 0.229, Fig. S6). Based on these data, our result suggests that CTB uptake is enhanced via increased GM1 expression due to the action of nSMase. 

**Fig. 5:**
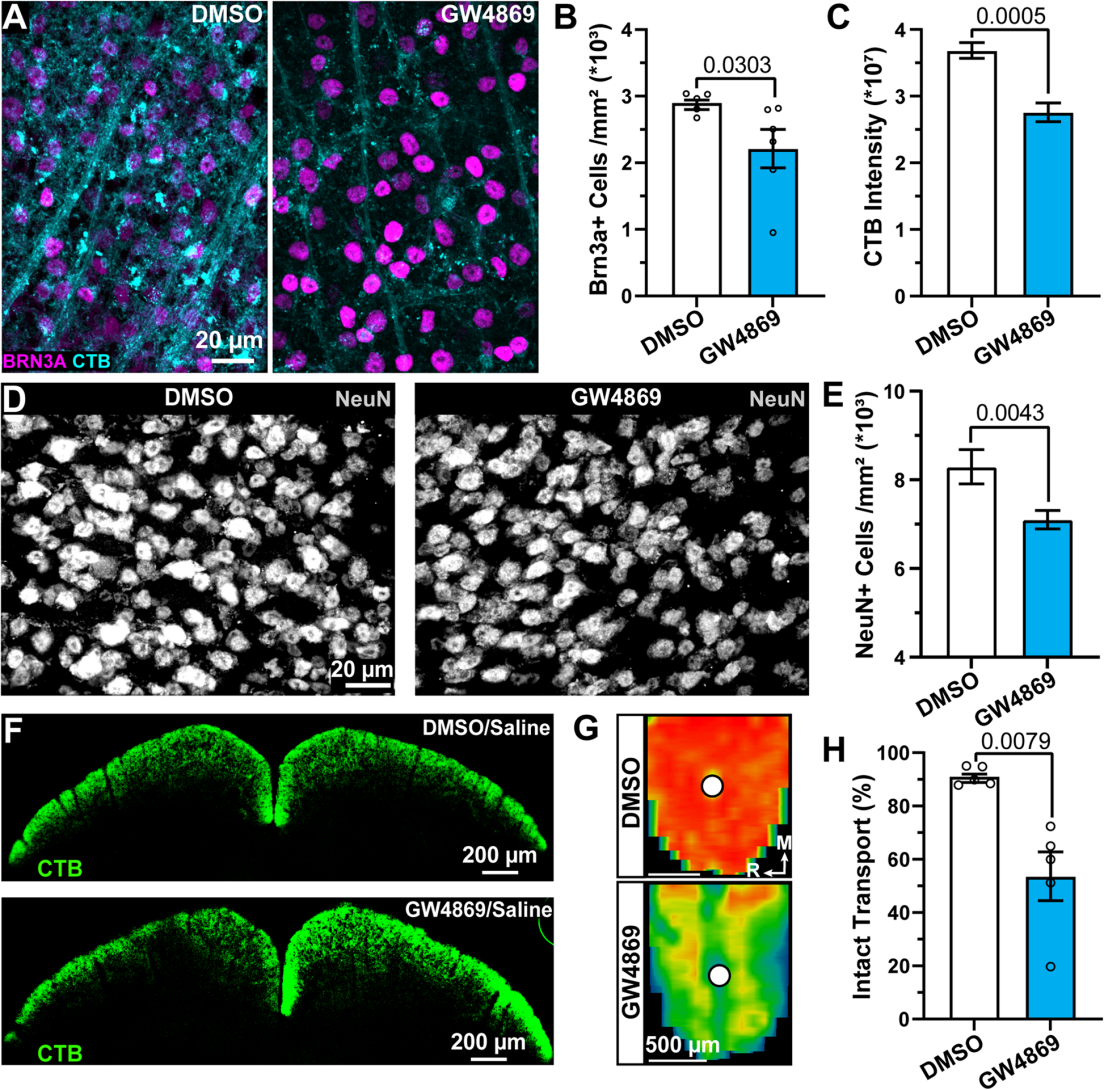
Prolonged exposure to GW4869 degrades CTB uptake and promotes visual tissue degeneration. **A** Example confocal micrographs from whole-mount retinas immunolabeled against BRN3A (magenta) following intravitreal injection of DMSO (left) or GW4869 for 20 days. Two days prior to endpoint, CTB (cyan) was intravitreally injected. **B** GW4869 significantly decreased density of BRN3A-positive RGCs (*p* = 0.0303) and (**C**) reduced CTB uptake by RGCs (*p* = 0.0005). *N* = 5 DMSO-treated retinas and 6 GW4869-treated retinas. **D** Example images of SC immunolabeled against NeuN following intravitreal injection of DMSO or GW4869. **E** GW4869 significantly reduced the density of NeuN-positive cells in the SC (*p* = 0.0043). *N* = 6 DMSO/saline treated animals and 9 GW4869/saline treated animals. (**F**, top) Confocal image of coronal section of superior colliculus (SC) following intravitreal injection of DMSO (left eye) or saline (right eye) and CTB. (**F**, bottom) Confocal images of SC following injection of GW4869 (left eye) or saline (right eye) and CTB. **G** Reconstructed heat map of fluorescence intensity of CTB in SC of a DMSO-treated eye (top) and a GW4869-treated eye (bottom). (**H**) GW4869 significantly blunted the percent of intact anterograde axonal transport of CTB to the SC (*p* = 0.0079). *N* = 5 DMSO/saline and 5 GW4869/saline animals. Statistics: (**B**, **C**, **E**) Mann–Whitney test, (**H**) *t*-test. Data presented as mean ± sem

Provided evidence that exosome supplementation produces neuroprotection in models of glaucoma and our findings that pharmacologically suppressing exosome generation increases apoptosis, we assayed the impact of intraocular injection of GW4869 on visual tissues in mice (Fig. 3) [15, 16]. We tested the influence of GW4869 on RGC and SC neuron density and RGC uptake and axonal transport of CTB to the SC. Eighteen days after a single intraocular injection of GW4869, we injected CTB into the eye. On day 20, we harvested tissues. At this time, CTB uptake by RGCs appeared diminished and RGC density seemed reduced (Fig. 5A). We found the density of BRN3A-positive RGCs significantly reduced by 23% in GW4869-injected eyes (2931 ± 71 vs. 2236 ± 291 cells/mm^2^, *p* = 0.0303, Fig. 5B). Quantification of CTB intensity in the RGC layer of whole-mount retinas demonstrated GW4869 significantly degraded CTB uptake by 40% compared to eyes injected with DMSO (*p* = 0.0005, Fig. 5C). To determine if GW4869 affected SC neuron viability, we counted the number of NeuN-positive cells in the SC. We found the density of NeuN-positive cells in the SC significantly reduced by 14% following intraocular injection of GW4869 compared to DMSO-injected eyes (8295 ± 385 vs. 7100 ± 205 cells/mm^2^, *p* = 0.0043, Fig. 5D, E). Similar to the reduction in CTB uptake by RGCs, RGC axon transport of CTB to the SC appeared reduced by GW4869 (Fig. 5F, G). As anticipated based on visual inspection of CTB fluorescence in the SC, GW4869 significantly reduced CTB intensity in the SC by 41% compared to DMSO-treated eyes, indicating deficits in anterograde axonal transport produced SC neuron dropout (*p* = 0.0079, Fig. 5H).

## Discussion

In this study we provide evidence that decreasing the biogenesis of putative exosomes by inhibiting nSMase, using GW4869, promotes degeneration in vitro and in vivo. In addition to reducing small EVs, inhibiting nSMase enhances the generation of larger EVs, including putative microvesicles and apoptotic bodies. The increase in larger EVs and their interactions with viable cells promotes degeneration. Our findings, in the context of developing treatments for neurodegenerations such as glaucoma, indicate that exosome supplementation may reduce disease progression [15, 16]. Moreover, our results suggest that harnessing particles that express apoptotic markers may also prove neuroprotective. These interventions may also increase the effectiveness of cell replacement therapies. Several studies provide evidence that donor hPSC- derived neurons have the potential to replace cells in host tissues lost to degeneration [45, 46]. However, studies also indicate that the percentage of donor cells that survive after transplantation is low [47]. Based on our data, we propose that transplantation efficiency might be increased by reducing pro-phagocytotic signals such as phosphatidylserine in donor cells.

Similar to our findings, others have previously shown that nSMase2 accumulates in RGCs derived from hPSCs and rodent RGCs in whole mount retinas, and incubating cells in GW4869 significantly reduces nSMase activity, which likely diminished nSMase2 immunolabeling in our hRGCs (Fig. 1A-C) [35, 48]. Notably, we found that short-term inhibition of nSMase by GW4869 produced a stronger effect on nSMase2 expression in vivo (-30%) compared to in vitro (-12%) (Figs. 1A-C and S2A, B). The more pronounced effect of GW4869 on nSMase2 in vivo indicates non-cell autonomous interactions. As expected, similar to the previous studies, we found that inhibition of nSMase by GW4869 reduced ceramide labeling in hRGCs (Fig. 1D-F) [35]. We suspect that inhibition of nSMase decreased the concentration of hydrophobic ceramide-rich platforms within plasma membranes [49]. Also, we observed that inhibition of nSMase enhanced expression of GM1 ganglioside but did not significantly affect GM1-associated ceramide (Fig. 1D-H). Others have found that SMase activity reduces the area of lipid rafts, and this reduction in area increases the density of smaller GM1 gangliosides [50]. Using the inverse pharmacologic approach of inhibiting nSMase activity, our data corroborated this finding.

Furthermore, one day after intraocular injection of GW4869, we observed increased GM1 in flat mount retinas (Fig. S2C, D), providing additional evidence that that inhibiting nSMase activity increases accumulation of GM1 ganglioside in the near term. This increase in GM1 ganglioside may be a neuroprotective compensatory response to the reduction in ceramide to maintain plasma membrane fluidity and potentiate responses to BDNF [26, 27, 51, 52]. If the latter is true, this compensatory GM1 enhancement does not require BDNF (exogenous BDNF was not included in our culture medium), but BDNF may be needed for short-term neuroprotection. Following prolonged exposure to GW4869 in vivo, we observed a reduction in CTB uptake by mouse RGCs and cell loss, suggesting that either the transient enhancement in GM1 does not provide enduring neuroprotection or simply CTB is reduced due to cell loss (Fig. 5A-C).

Several investigations have provided evidence that inhibiting nSMase activity decreases the biogenesis of exosomes produced through the ceramide-dependent pathway [11, 25, 53, 54]. Similarly, here, we found that GW4869 produced a 90 nm increase in the peak density of extracellular particle size in the 100,000 × g sediment (Fig. 2B, C). This finding suggests that the 100,000 × g pellet from DMSO-treated cells contained exosomes. However, differential centrifugation alone may not isolate a pure collection of exosomes [55, 56]. In future studies, we will attempt to increase the purity of exosomes by implementing the cushion-density gradient ultracentrifugation strategy [55].

A previous report indicated that inhibition of nSMase activity by application of GW4869 or nSMase knockdown not only reduced the concentration of small EVs, but also enhanced the concentration of large EV generated by outer plasma membrane blebbing [12]. Here, we provide additional proof for this phenomenon. Our data indicate that GW4869 significantly enhanced the generation of large extracellular particles in both the 10,000 and 100,000 × g sediments (Fig. 2B, C). This result suggests that our 100,000 × g sediment from GW4869-treated cells also contained microvesicles. In addition to microvesicles, this sediment contained protein labeling for ANXA5, which is an indicator of apoptotic bodies [1]. Thus, GW4869 appears to not only reduce the biogenesis of exosomes but also increases the concentration of microvesicles and apoptosis-related particles in the 100,000 × g sediment.

Increased SMase activity is related to several diseases, and many studies have found that stress-related stimuli evoke SMase activity that promotes ceramide-dependent apoptosis [57–60]. However, here, we found inhibition of nSMase activity by GW4869 significantly increased apoptosis based on ANXA5 western blot and on pSIVA labeling (Figs. S3; 3A,B). This finding likely indicates that a disturbance in lipid metabolism and EV milieu will have detrimental effects on cell viability regardless if sphingolipid metabolism is increased or reduced [61].

Interestingly, when we exchanged the original medium for fresh medium, removing many of the apoptotic particles, in the GW4869-treated cultures, we observed on-going apoptosis (i.e., increased pSIVA intensity over time) was abolished (Fig. 3A-C). This finding suggests that apoptotic particles promote apoptosis. However, based solely on this set of experiments, it remains unclear if the increase in larger EVs, reduction in smaller EVs, or reconfiguration of membrane-associated lipids produced by GW4869 treatment increased apoptosis. Despite these short-comings, we provide visual evidence that inhibition of nSMase increases apoptosis, and interestingly, apoptotic particles invade or adhere to viable cells promoting Wallerian-like degeneration (Fig. 3D, E) [19, 62].

Other investigators have reported intercellular trafficking of mitochondria via EVs that functionally integrate into the target cell [43, 63, 64]. Our data also provide proof for physiologic cell-to-cell trafficking of mitochondria. Using our coculture approach (Fig. 4A), we found cells donate mitochondria to neighboring cells, and in some images, we noticed mitochondria in the extracellular space (Fig. 4C). Compared to intracellular mitochondria in treated and naïve cells, mitochondria in the extracellular space appeared smaller, suggesting these might be degraded mitochondria ejected from cells that would normally be removed by phagocytes in vivo [65]. Application of GW4869 reduced mitochondrial-labeling in cells directly treated, and enhanced accumulation of mtSOX in both treated and cocultured naïve cells (Fig. 4C-F). Therefore, we predict that GW4869 decreased the number of viable mitochondria that could be labeled by MitoTracker. Moreover, our data indicates that inhibition of nSMase by GW4869 enhanced mitochondria labeled by mtSOX not only in cells directly treated but also in cocultured naïve cells. These results suggest that perturbing the metabolism of sphingolipids influences not only the cells treated with GW4869 but also naïve cells in the coculture. In future experiments, we will determine if the uptake of mitochondrial reactive oxygen species by naïve cells impacts viability.

Finally, we determined the influence of GW4869 on RGC and SC neuron density and RGC axon transport to the SC. Inhibition of nSMase by GW4869 reduced RGC bodies in the retina and the density of neurons in the SC (Fig. 5A-E). This frank degeneration is likely subsequent to degradation of RGC axon transport, which was 2-3X more dramatic, similar to the progression of glaucomatous axonopathy (Fig. 5F-H) [19, 21]. Our findings indicate that inhibition of nSMase by GW4869 produces both near-term effects on RGC density and axon transport that significantly effects upstream SC neuron density. Taken together, we predict that localized treatment with GW4869 reduces exosomes and increases large apoptotic particles, promoting RGC degeneration, and SC neurons are lost due to deficits in RGC axon function.

### Supplementary Information

**Additional file1: Movie 1. **tdTomato-expressing hRGCs labeled with pSIVA in control medium. **Movie 2.** hRGCs labeled with pSIVA following 3 h in GW4869. **Movie 3.** hRGCs labeled with pSIVA after 24 h in GW4869. **Movie 4.** DMSO treated hRGCs labeled with MitoTracker and CTB cocultured with naïve cells. **Movie 5.** GW4869 treated hRGCs labeled with MitoTracker and CTB cocultured with naïve cells. **Figure S1.** Confirmation of nSMase2 antibody selectivity and ceramide localization. (A) Example confocal images show punctate expression and localization of nSMase2 immunolabeling in the soma of hRGCs. (B) hRGCs incubated with only the appropriate secondary antibody shows the selectivity of the nSMase2 antibody. (C) Incubation of hRGCs with the fluorescently labeled C6-NBD ceramide shows localization of ceramide to the soma of hRGCs, **Figure S2.** Intraocular injection of GW4869 reduces nSMase2 and enhances GM1 immunofluorescence in the RGC layer of whole-mount retinas. (A) Confocal images of whole-mount mouse retinas following intraocular injection of either DMSO (left) or 10 µM GW4869 (right) immunolabeled against RBPMS (green) and nSMase2 (magenta). (B) After 24 h of DMSO or GW4869 treatment, nSMase2 immunofluorescence significantly decreased (p = 0.0444). N = 3 retinas for each condition from 3 mice. (C) Example images of mouse whole-mount retinas labeled for GM1 ganglioside (red), TUJ1 (green), and RBPMS (blue) one day after intraocular DMSO or GW4869. Insets show orthogonal rotations of each en face image. GW4869 significantly increased GM1 labeling in the RGC layer (p = 0.0237).  Statistics: Paired t-tests. **Figure S3.** GW4869 treatment increases ANXA5 expression in small EVs from hRGCs. Western blot image shows an increase in band size from small EVs collected from GW4869 treated hRGCs compared to controls. Analysis of the western blot shows a significant increase in ANXA5-positive apoptotic bodies in small EVs following treatment with GW4869 (*p=0.0115, n=2). Statistics: t-test. **Figure S4.** Confirmation of transition from apoptotic initiation to end-stage apoptosis. Example confocal micrograph shows the overlap in signal of pSIVA (green) and propidium iodide (PI, magenta) over time in ARPE-19 cells, indicating the progression from the early initiation of apoptosis to end stage apoptosis. **Figure S5.** Confirmation of intercellular mitochondrial transfer. After 24 h in vitro, tdTomato-expressing hRGCs (orange) were treated with CTB-647 (Magenta) and CellLight Mitochondria-GFP (Mito, green). After repeated medium exchanges to remove residual CTB and Mito labeling, naïve tdTomato-expressing hRGCs, without CTB-647 and Mito labeling, were cocultured. After 24 h in coculture, samples were imaged by confocal microscopy. **Figure S6.** Inhibition of aSMase does not influence mitochondria or CTB labeling. (A) Example confocal images of CTB-labeled (green) tdTomato-positive cells (orange) with mitochondria labeled with MitoTracker (magenta) directly treated with desipramine and cocultured with naïve cells (tdTomato-labeling with sparse CTB labeling). (B) Inhibition of aSMase with desipramine did not significantly affect mitochondrial labeling in treated (p > 0.9999) or naïve cells (p = 0.5054). (C) Desipramine did not significantly influence CTB uptake in cells directly treated (p > 0.9999) or in cocultured naïve cells compared to controls (p = 0.229). N = 25 cells directly treated with desipramine and 25 naïve cells from 3-4 replicates. Statistics: Kruskal-Wallis tests.

## Data Availability

The datasets used and analyzed during the current study are available from the corresponding authors on reasonable request.
