# Supplementary material for: Neutral sphingomyelinase inhibition promotes local and network degeneration in vitro and in vivo
Source: Cell Commun Signal. 2023 Oct 30;21:305. doi: 10.1186/s12964-023-01291-1 (PMC10614343; doi:10.1186/s12964-023-01291-1)

**Movies and Supplement Materials**

**Movie 1. tdTomato-expressing hRGCs labeled with pSIVA in control medium.**

**Movie 2. hRGCs labeled with pSIVA following 3 h in GW4869.**

**Movie 3. hRGCs labeled with pSIVA after 24 h in GW4869.**

**Movie 4. DMSO treated hRGCs labeled with MitoTracker and CTB cocultured with naïve cells.**

**Movie 5. GW4869 treated hRGCs labeled with MitoTracker and CTB cocultured with naïve cells.**

**Figure S1. Confirmation of nSMase2 antibody selectivity and ceramide localization. (A)** Example confocal images show punctate expression and localization of nSMase2 immunolabeling in the soma of hRGCs. **(B)** hRGCs incubated with only the appropriate secondary antibody shows the selectivity of the nSMase2 antibody. **(C)** Incubation of hRGCs with the fluorescently labeled C6-NBD ceramide shows localization of ceramide to the soma of hRGCs,

**Figure S2. Intraocular injection of GW4869 reduces nSMase2 and enhances GM1 immunofluorescence in the RGC layer of whole-mount retinas.** (**A**) Confocal images of whole-mount mouse retinas following intraocular injection of either DMSO (left) or 10 µM GW4869 (right) immunolabeled against RBPMS (green) and nSMase2 (magenta). (**B**) After 24 h of DMSO or GW4869 treatment, nSMase2 immunofluorescence significantly decreased (p = 0.0444). N = 3 retinas for each condition from 3 mice. (**C**) Example images of mouse whole-mount retinas labeled for GM1 ganglioside (red), TUJ1 (green), and RBPMS (blue) one day after intraocular DMSO or GW4869. Insets show orthogonal rotations of each *en face* image. GW4869 significantly increased GM1 labeling in the RGC layer (p = 0.0237). Statistics: Paired *t*-tests.

**Figure S3. GW4869 treatment increases ANXA5 expression in small EVs from hRGCs.** Western blot image shows an increase in band size from small EVs collected from GW4869 treated hRGCs compared to controls. Analysis of the western blot shows a significant increase in ANXA5-positive apoptotic bodies in small EVs following treatment with GW4869 (*p=0.0115, n=3). Statistics: *t*-test.

**Figure S4. Confirmation of transition from apoptotic initiation to end-stage apoptosis.** Example confocal micrograph shows the overlap in signal of pSIVA (green) and propidium iodide (magenta) over time in ARPE-19 cells, indicating the progression from the early initiation of apoptosis to end stage apoptosis.

**Figure S5. Confirmation of intercellular mitochondrial transfer.** After 24 h in vitro, tdTomato-expressing hRGCs (orange) were treated with CTB-647 (Magenta) and CellLight Mitochondria-GFP (Mito, green). After repeated medium exchanges to remove residual CTB and Mito labeling, naïve tdTomato-expressing hRGCs, without CTB-647 and Mito labeling, were cocultured. After 24 h in coculture, samples were imaged by confocal microscopy.

**Figure S6. Inhibition of aSMase does not influence mitochondria or CTB labeling.** (**A**) Example confocal images of CTB-labeled (green) tdTomato-positive cells (orange) with mitochondria labeled with MitoTracker (magenta) directly treated with desipramine and cocultured with naïve cells (tdTomato-labeling with sparse CTB labeling). (**B**) Inhibition of aSMase with desipramine did not significantly affect mitochondrial labeling in treated (p > 0.9999) or naïve cells (p = 0.5054). (**C**) Desipramine did not significantly influence CTB uptake in cells directly treated (p > 0.9999) or in cocultured naïve cells compared to controls (p = 0.229). N = 25 cells directly treated with desipramine and 25 naïve cells from 3-4 replicates. Statistics: Kruskal-Wallis tests.

**Supplemental Figures**

**Figure S1**

**
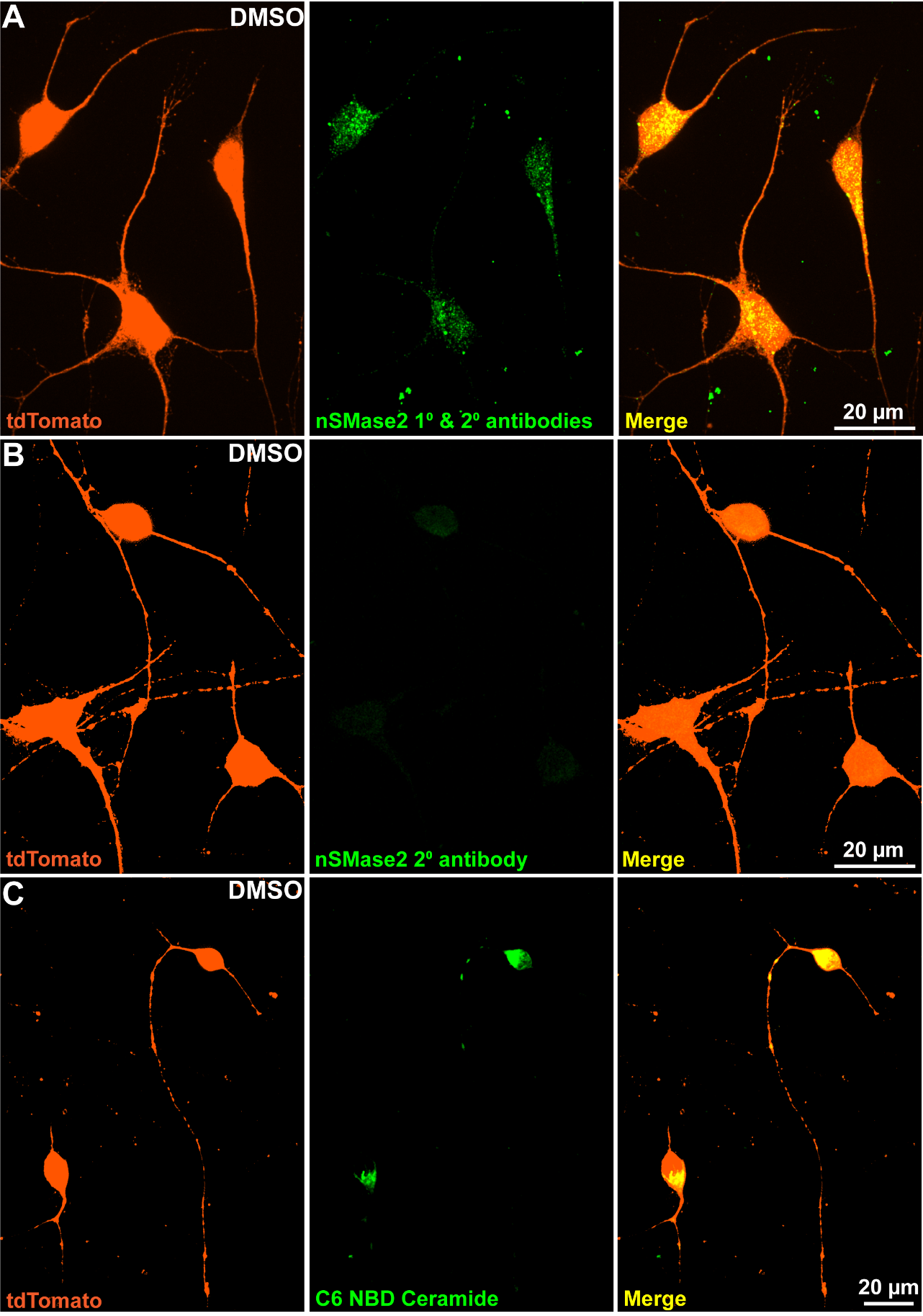
**

**Figure S2**


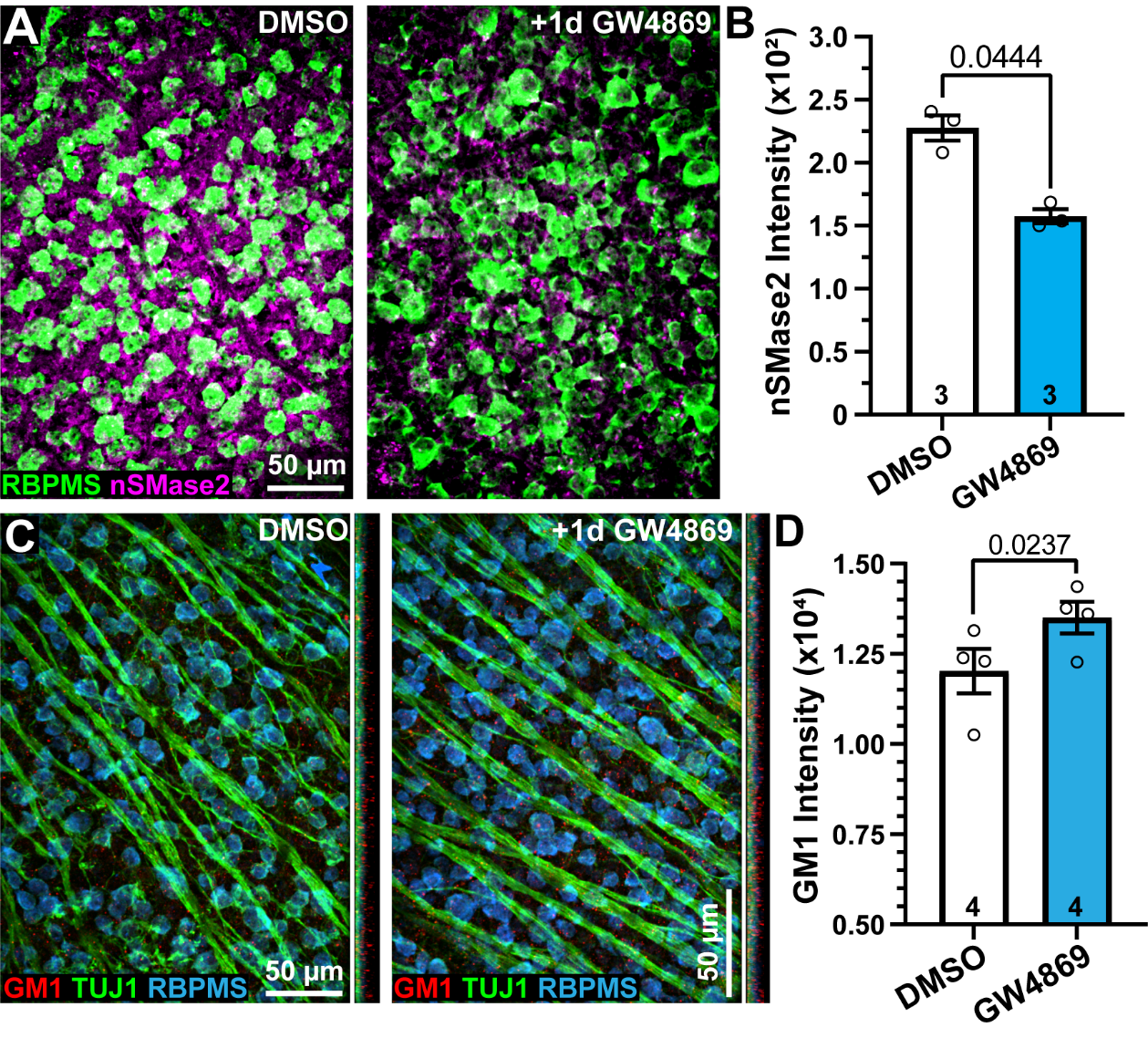


**Figure S3**

**
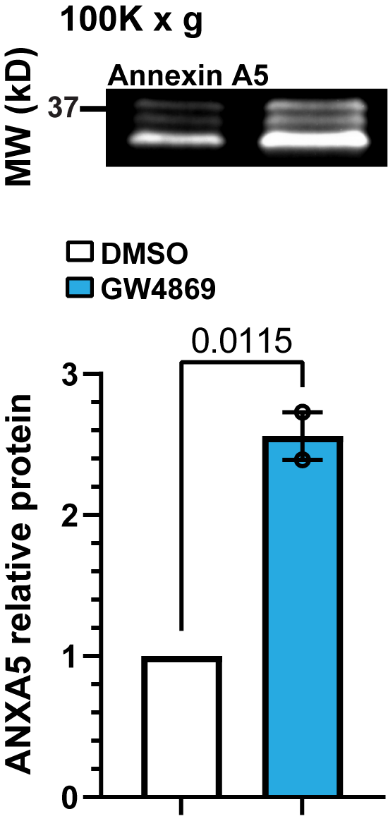
**

**Figure S4**

**
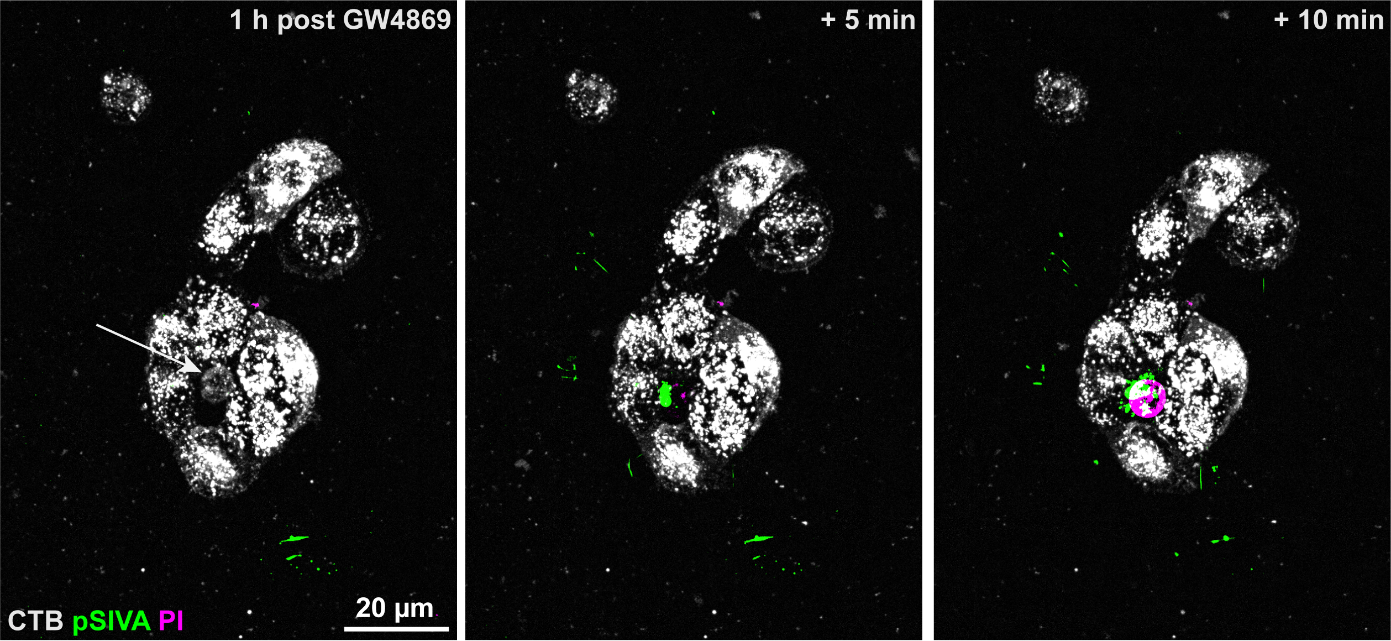
**


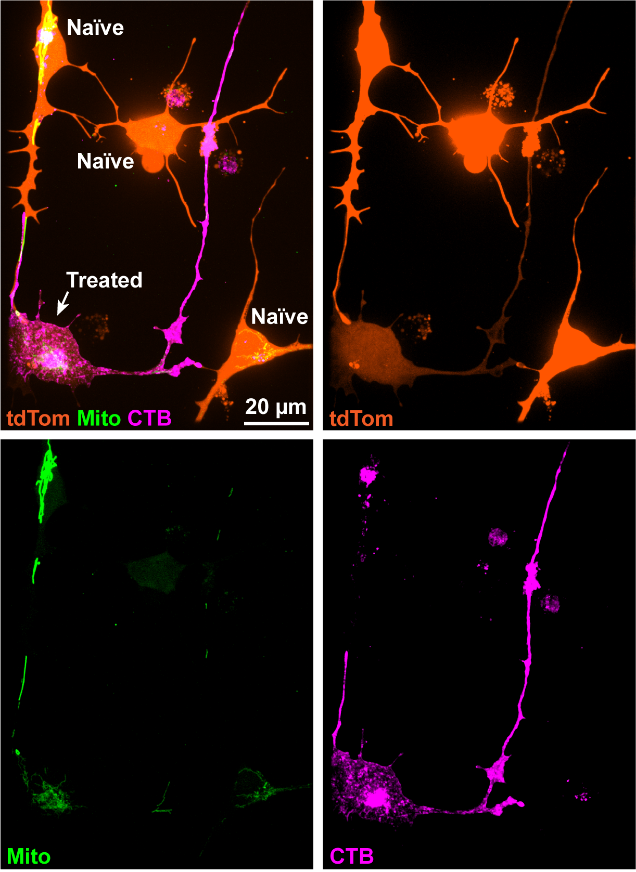
**Figure S5**

**Figure S6**


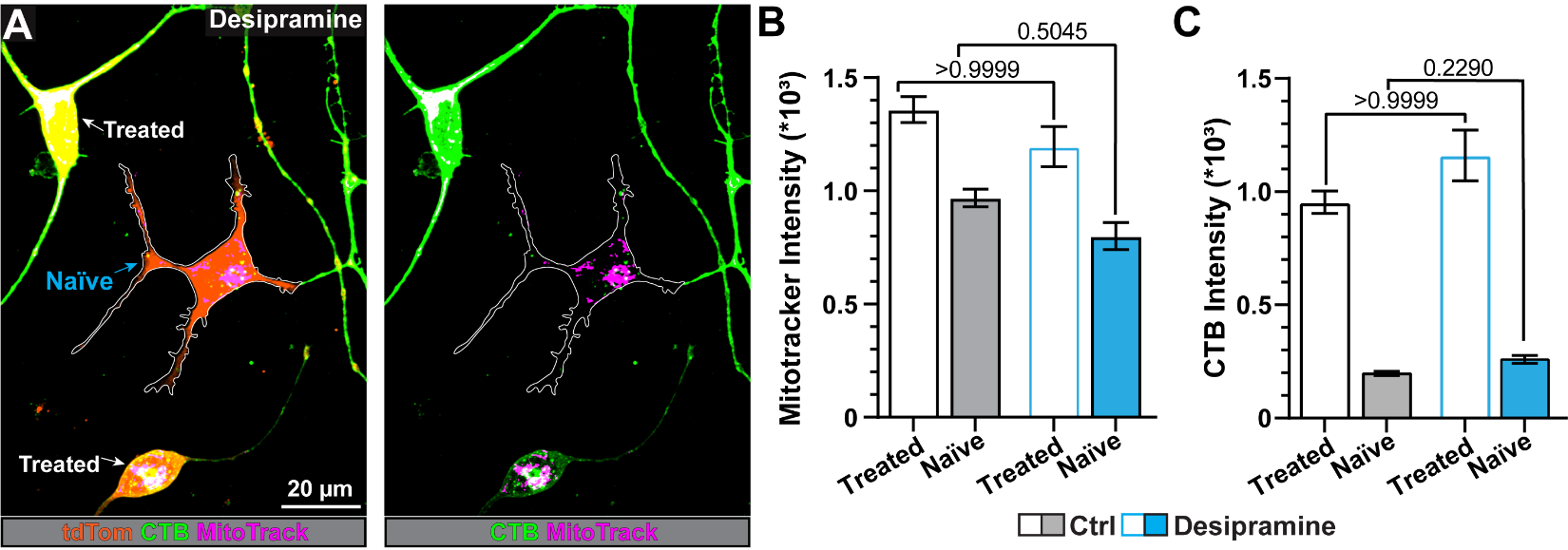

Supplement: Supplementary file 2 — Additional file1: Movie 1. tdTomato-expressing hRGCs labeled with pSIVA in control medium. Movie 2. hRGCs labeled with pSIVA following 3 h in GW4869. Movie 3. hRGCs labeled with pSIVA after 24 h in GW4869. Movie 4. DMSO treated hRGCs labeled with MitoTracker and CTB cocultured with naïve cells. Movie 5. GW4869 treated hRGCs labeled with MitoTracker and CTB cocultured with naïve cells. Figure S1. Confirmation of nSMase2 antibody selectivity and ceramide localization. (A) Example confocal images show punctate expression and localization of nSMase2 immunolabeling in the soma of hRGCs. (B) hRGCs incubated with only the appropriate secondary antibody shows the selectivity of the nSMase2 antibody. (C) Incubation of hRGCs with the fluorescently labeled C6-NBD ceramide shows localization of ceramide to the soma of hRGCs, Figure S2. Intraocular injection of GW4869 reduces nSMase2 and enhances GM1 immunofluorescence in the RGC layer of whole-mount retinas. (A) Confocal images of whole-mount mouse retinas following intraocular injection of either DMSO (left) or 10 µM GW4869 (right) immunolabeled against RBPMS (green) and nSMase2 (magenta). (B) After 24 h of DMSO or GW4869 treatment, nSMase2 immunofluorescence significantly decreased (p = 0.0444). N = 3 retinas for each condition from 3 mice. (C) Example images of mouse whole-mount retinas labeled for GM1 ganglioside (red), TUJ1 (green), and RBPMS (blue) one day after intraocular DMSO or GW4869. Insets show orthogonal rotations of each en face image. GW4869 significantly increased GM1 labeling in the RGC layer (p = 0.0237). Statistics: Paired t-tests. Figure S3. GW4869 treatment increases ANXA5 expression in small EVs from hRGCs. Western blot image shows an increase in band size from small EVs collected from GW4869 treated hRGCs compared to controls. Analysis of the western blot shows a significant increase in ANXA5-positive apoptotic bodies in small EVs following treatment with GW4869 (*p=0.0115, n=2). Statistics: t-test. Figu [file 12964_2023_1291_MOESM1_ESM.zip › Movies and Supplementary Materials REV.docx]
